# Tracing 25 years of ‘initiativitis’ in central government attempts to join up local public services in England

**DOI:** 10.1332/030557321X16837266852569

**Published:** 2023-06-16

**Authors:** Michael Gibson, Felix-Anselm van Lier, Eleanor Carter

**Affiliations:** University of Oxford, UK

**Keywords:** joined-up public services, central–local relations, governance, accountability, public sector reform, central government, UK

## Abstract

Over the last 25 years, central government has attempted to join up local public services in England on at least 55 occasions, illustrating the ‘initiativitis’ inflicted upon local governments by the large volume and variety of coordination programmes. By analysing and mapping some of the characteristics of these initiatives, we have uncovered insights into the ways central government has sought to achieve local coordination. We observe a clear preference for the use of funding and fiscal powers as a lever, a competitive allocation process, and a constrained discretion model of governance, with some distinct patterns over time. These choices made in the design of initiatives are likely to be shaped by the perceived and real accountability structures within government, and so offer an opportunity to consider how accountability affects, and is affected by, particular programmatic efforts at a local level.

This article makes a significant contribution to our understanding of coordination programmes at a central–local government level. By identifying patterns in the approach of government over the last 25 years, it offers an empirical lens to map the ‘glacial and incremental’ reframing of central–local relations and associated shifts in public accountability. In this way, the article provides more solid foundations to a range of issues – central government’s reliance on controlling the reins of funding, the competitive nature of allocation processes, and the enduring centralisation of accountability – that have been much discussed among policymakers, practitioners and researchers, but have lacked clear empirical grounding.

## Introduction

Many of the most complex social issues do not easily map onto the structures of public service delivery (Wilson et al, 2015; OECD, 2017). An individual facing long-term unemployment might also experience insecure housing or homelessness, mental health conditions, and substance misuse issues, but support is often divided between different parts of government and independent provider organisations (Bramley and Fitzpatrick, 2015). Since 1997, successive central government administrations in England have pursued a range of initiatives to improve the local coordination of public services for cross-cutting issues (Wilson et al, 2015). The rhetoric and practice of these efforts to ‘join up’ government transcends partisan divides, as the centralised English state has sought to respond to the multi-dimensional fragmentation of public services (Elliott et al, 2022), and the resulting failures to make efficient use of public resources and meet the needs of service users (Bramley and Fitzpatrick, 2015; Peters, 2018). However, the concept of joining up, the mechanisms through which it can be achieved, and its potential to effectively address fragmentation is less clear.

Dunleavy (2013) notes that while ‘joining up’ has been a theme in practitioner discussions for many years, academic research is more limited. The literature exploring issues of joining up to date has focused on distinct angles. Some authors have focused on joining up (for example, Pollitt, 2003; Bogdanor, 2005), or its variations such as ‘holistic governance’ (6 et al, 2002), as a broad theoretical concept. Others have sought to explore how the idea has evolved over time (Hood, 2005) and been applied to different levels of government (Stoker, 2005; Catney, 2009). However, this conceptual work often lacks substantive empirical foundation. The available patchwork of small-scale empirical work tends to focus on particular policy areas, or even individual programmes (Dunleavy, 2013; Hudson, 2019). Indeed, a large part, if not most, of our empirical understanding of programmes that seek to integrate social services comes from evaluations that were attached to each of these programmes (for example, the New Deal for Communities evaluation, Batty et al, 2010). We offer an operationalisation of Hood and Margetts (2007) and Stoker (2005) frameworks that instead supports conceptual advances with corresponding empirical work, adopting ‘joining up’ as our boundary condition. This is particularly important as initiatives often implicitly or explicitly seek to bridge or dissolve traditional policy boundaries, limiting the utility of aforementioned policy- or programme-specific empirics.

In this article, we unpack and comparatively describe the strategic choices around programme management made by central government in England in efforts to improve local public service coordination.^1^ In particular, we focus on the ways in which central governments have used the resources at their disposal to leverage change, the processes through which particular areas were chosen for support, and the governance model adopted in each programme. In doing so, we explore the relationship between these choices and models of public accountability, unpacking the tension between the traditional, hierarchical ‘Westminster’ model and a multi-level, multi-party policy landscape (Mulgan, 2014; Papadopoulos, 2014; Matthews and Flinders, 2017; Beel et al, 2021). We examine the evolving public accountability landscape by tracing a variety of different approaches through which central government creates initiatives (Hood, 1983; Hood and Margetts, 2007), drawing on legal reform, funding arrangements and central government officials directly or indirectly influencing practice, in an attempt to reshape and reform local public service delivery. We also seek to understand the different local governance models through which central government attempts to coordinate local public services in the context of a ‘hollowed-out’ local government (Skelcher, 2000). The result is a variety of models which central government may pursue, offering varying levels of autonomy to local institutions in response to a perceived or actual lack of capacity in local government (Stoker, 2005). We bring together these two theoretical frameworks which capture both the levers applied centrally and the effect of these levers on local autonomy, as well as the way in which schemes were geographically allocated, to trace the various approaches to achieving join-up and how programme design decisions are shaped by the public accountability landscape.

This article focuses on the high-level, *ex-ante* policy content of joining up initiatives. As a number of scholars have argued, this alone cannot fully illuminate the policy process (for example, Sausman et al, 2016; Eckersley and Tobin, 2019). However, to support more synthetic work, this article attempts to map the programmes which provide the link between high-level ambition and on-the-ground delivery. In doing so, we inevitably leave some elements – from the rationale of central government to the realities of frontline implementation through to the ultimate outcomes of the policy – underdeveloped. We therefore hope that this exploratory piece will be followed by further research which encompasses local joining up policy ‘from signals of intent to the final outcomes’ (Cairney, 2016: 2).

Nevertheless, the article offers a number of contributions to existing scholarly work: we blend and then operationalise two established theoretical frameworks to unpack the mechanism of joining up (the third section), and analyse 55 policy initiatives between 1997 and 2022 against this schematic (the fourth section). In doing so, we identify patterns in the approach of government over the last 25 years (the fifth section), offering an empirical lens to map the ‘glacial and incremental’ reframing of central–local relations (Sandford, 2019: 8) and associated shifts in public accountability. We thereby provide more solid foundations to a range of issues – central government’s reliance on controlling the reins of funding, the competitive nature of allocation processes, and the enduring centralisation of accountability – that have been much discussed among policymakers, practitioners and researchers, but have lacked clear empirical grounding (the sixth section). We explore how these issues relate to central government’s understanding of public accountability, considering how the limited efforts to decentralise control map against Hooghe and Marks’ (2003) typology of multi-level governance, and set out a future research agenda to examine the evolving central–local public accountability landscape (the seventh section). In the following section, we explore the varying concepts and motivations that provide the backdrop to 25 years of efforts to join up local public services.

## Joining up local public services

Historically, local governments in England oversaw expanding public services in their jurisdictions in relative autonomy from central government and with considerable administrative discretion over the implementation of national policies (John, 2014: 690; Leach et al, 2017). However, the emergence of a national welfare state saw this autonomy diminish, with the absorption of local institutions and capacities into the ‘apparatus of the central state’ (Loughlin, 1996: 72) as part of the consistent reconfiguration and rescaling of the administrative landscape since the Second World War (Bradbury, 2007; Ayres et al, 2017). Additionally, marketisation saw those services which remained in the purview of local government increasingly outsourced to private and third sector organisations, resulting in a complex and fragmented public service landscape characterised by growing numbers of service provider organisations and increased competition between them (Sasse et al, 2019; Barnett et al, 2021). Adding to this complexity, central government responsibility is also split horizontally, leading to the persistent challenge of ‘departmentalism’ or ‘siloed’ policymaking (Albrow, 1970: 88; Hood, 1976: 17–20; Peters, 2018).

Joining up at a local level seeks to address this fragmentation of local service capacity. While coordination has been a long-running concern for governments (Peters, 2018), it gained particular traction in the UK under the New Labour government, which spelled out a strategy to integrate ‘policies and programmes, local and national’ in a ‘joined up way’ in its Modernising Government white paper (Cabinet Office, 1999; see also Clark, 2002; Bogdanor, 2005). Since then, the rhetoric of joined-up policymaking has been gradually absorbed into ‘the culture of Whitehall’ (Bogdanor, 2005: 17) – every central government administration over the last 25 years has adopted policies introducing some form of ‘joining up’, ‘service integration’, ‘systems thinking’, ‘localism’, ‘devolution’, ‘local collaboration’ or ‘place-based partnerships’, each of which (although often ambiguously) emphasises some element of local public service coordination (for example, Pollitt, 2003; Bogdanor, 2005; Sandford, 2022).

The varied terminology runs parallel to a varied set of justifications for improving the coordination of policies (Pollitt, 2003; Peters, 2018). Often, there is an implicit assumption that joining up will improve public service performance, but what this ‘improved performance’ represents might encompass improvements in one or more facets of value for money, either aspiring to economy, efficiency, effectiveness, equity, service quality, or some combination of these (Boyne, 2003). Over the period of our study, it is likely that all of these rationales play some part in underpinning particular efforts to join up local complex public services, to different extents at different times.

Reforms which seek to join up public services provide a useful lens through which to explore broader ideas of central–local relations and public accountability. They are linked – both implicitly through their connection to concepts like ‘place-based working’ (Taylor and Buckly, 2017), and explicitly through devolution policy (for example, Sandford, 2022; Ward, 2022 – although the devolution project encompasses a much wider set of policy reforms beyond service coordination) – to the broader rhetoric and policy programme of decentralisation in England. In the context of local public service coordination, this often originates from a functionalist logic, whereby it is believed that ‘every public good has an optimal spatial scale’, as opposed to moral demands for self-rule (Hooghe et al, 2020: 194). This tallies with the perception that it is often ‘easier to secure joined-up government at the local level than in Whitehall’ (Bogdanor, 2005: 12) – although it is worth noting that even this dimension of localism is not entirely without controversy (Fitzpatrick et al, 2020). Hence, the focus of this study is on the design of managerial elements of programmes, rather than federal innovations.

The set of initiatives we explore in this article also offers a concrete and bounded frame to explore broader concepts. In particular, they highlight a growing tension between the traditional approach to public accountability in England and an increasingly complex public service system. Public accountability in England has long followed the ‘Westminster’ model, in which civil servants are hierarchically accountable to their ministers, who are in turn accountable to parliament. Parliament, finally, is accountable to the broader public via the ballot box (Barberis, 1998). This perception of accountability remains dominant among politicians and civil servants (for example, House of Lords Constitution Committee, 2012; Paun and Barlow, 2013), and hence remains influential in the way central government designs and manages programmes.

‘Multi-level governance’ is increasingly used to describe a landscape in which authority is distributed between different actors and levels of government (Hooghe et al, 2020). Beyond Whitehall, but also within it, the insufficiencies of the Westminster model have been widely recognised. Even in cases where central government remains the sole actor, public servants are increasingly held directly accountable by outside bodies – parliamentary committees, ombudsman services, courts – suggesting a pluralist mode of accountability may have supplanted the Westminster model (Mulgan, 1997). This is magnified in cases, such as those examined in this article, where central government interacts with or operates through actors at a subnational level. Here, fractured responsibilities across multiple levels create further pressure to move away from a single, hierarchical line of accountability towards multi-level governance (Papadopoulos, 2014; Hooghe et al, 2020), which itself might take a range of forms spanning from holistic federalism to narrow, programme-specific arrangements (Hooghe and Marks, 2003). As a result, the understanding of accountability within central government appears to sit in tension with the de facto accountability arrangements created or demanded by their policy approach.

## Programme management choices in joining up from central to local

As described in the previous section, initiatives seeking to join up local public services sit at the intersection of a wide range of concepts – some squarely focused on the need to better connect and coordinate services, others sitting within much broader debates surrounding the relationship between central and local government and governance. While many of these concepts are related, they themselves do not neatly ‘join up’ with one another. In order to lay the foundations for future research, then, we seek to map these initiatives, and the approach they adopt to achieve local public service join up.

We focus on three dimensions of initiatives to connect central government design and decision-making to the intended local approach, namely the levers of central government to initiate local joining up, the allocation processes used to enable local joining up, and the espoused governance model for local actors to participate in joining up. We examine several strategic choices in the management of a diverse set of centrally-driven initiatives cutting across a range of policy areas, united by an aspiration to improve coordination at a local level. In doing so, we also hope to lay the groundwork for further evaluation of the success of local joining up initiatives (see the seventh section). To this end, we have adapted established public administration theoretical frameworks, in line with consultation with policymakers and practitioners, in order to classify different aspects of the initiatives through which central government has sought to join up local public services.

### The central government lever

There are several ways in which a policy can be enacted in order to influence local practice, reflecting the options available to government more broadly. There have been a number of attempts to classify these levers (for example, Falleti, 2005; Wilson et al, 2015). Here, we focus in particular on the *Tools of Government* (Hood, 1983; Hood and Margetts, 2007). In operationalising this framework to explore central efforts to initiate local join-up, we adopt broadly the same classification as Hood and Margetts (Table 1). Our point of deviation is to collapse nodality and organisation into a single category: administration. Organisation, the ability for the people and equipment under central government’s direct control to effect change, is of limited application when trying to alter the structure of public services at a sub-national level. Where it is applied, it is most likely to function indirectly as nodality, with a new central team essentially tasked with cajoling local actors into reform. In cases where there is no clear regulatory reform or funding arrangement, it is generally difficult to unpack which of these elements is intended to initiate change, beyond the fact that central government officials either directly or indirectly seek to alter local practice.

Often, policies may exhibit multiple, overlapping levers. However, we sought to identify the primary lever through which change is initiated. As a result, we adopt a hierarchical classification system, focusing on the burden to government implied by a particular lever. Hence where elements of law and regulation are present – which often require significant effort on the part of government, such as passing an Act of Parliament – the initiative is classified as such, regardless of whether elements of funding and fiscal powers are also present. Where neither law and regulation nor funding and fiscal powers are present, the initiative is classified as administration. Effectively, administration represents a ‘low-cost’ option for the centre – in contrast to the more significant costs associated with either allocating funding or enacting legislative change.

### The allocation process

In addition to understanding the levers used by central government to initiate change in the local public services landscape, we identified the allocation process used to select the recipients of a particular initiative. We explore how initiatives are allocated to places, building on typologies of allocation processes for local authority funding (Smith, 2007; NAO, 2021). Some initiatives are universal, applying to the whole of England (or the United Kingdom, including England), while others are targeted at particular local areas, according to different criteria.

How localities are selected to receive support (or not) carries important implications for the relationship between central government and local actors. The use of competitive processes to allocate central government funding has been widely decried by those in local government. Andy Burnham, Mayor of Greater Manchester, criticised the ‘bidding culture’ cultivated by central government in its approach to local funding as ‘wrong on every level’ (Institute for Government, 2022). However, particular allocation processes might offer greater perceived control to central government. For example, targeting on the basis of competition, where recipients are scored against centrally-determined criteria, may allow government officials to exert more influence over the direction of local actors.

### The governance model

Finally, we have sought to understand the intended role of local actors in efforts to join up local public services. In order to do so, we have operationalised Stoker’s (2005) ‘two models of governance’ (along with a third model of top down, central governance suggested in the same chapter) for joined-up government programmes at a local or regional level. In order to analyse the aspired governance model in each initiative, we assess two dimensions: the level at which objectives are determined and accountability ultimately sits, and the level at which the approach to achieving those objectives is determined.

### Objective setting/political accountability

The first dimension on which to differentiate governance models is the level of government at which objectives are set, and hence to which those responsible for achieving the objectives are accountable. Ultimately, even the most ambitious devolution programmes in England to date have remained accountable to central government (Henderson et al, 2023). Nevertheless, even if ultimate accountability seems likely to remain at the centre, a number of initiatives seek to transfer at least immediate political accountability (Cendon, 2000; Christie, 2018) for the achievement of objectives to the local level.

### Approach to achieving objectives

The second dimension is the level at which the delivery approach is determined. Often, even if the objectives of an initiative are determined centrally, significant autonomy over *how* those objectives are achieved may be given to local actors.

Stoker describes traditional Westminster government policymaking as tending to adopt a top-down approach of command and control, in which central government imposes clear requirements for what it expects to be delivered, how it expects it to be delivered, and measures performance, in the model of New Labour’s ‘Barberism’. However, concerns with this model tend to focus on its ‘one-size-fits-all’ approach, which fails to account for local variation (Stoker, 2005: 165). In response, Stoker explores two alternative, emerging governance models. Community leadership views local government as the steward responsible for articulating and delivering community objectives, either directly or in partnership with a wider group of cross-sector local actors (Stoker, 2005: 162). Constrained discretion, meanwhile, sits somewhere in between these two poles, with ‘a decentralised image of management but a centralised image of politics’ (Stoker, 2005: 166). Accountability lines run to Westminster, with local actors offered some flexibility to deliver on a central agenda.

## Methodology and data

Using an iterative process, we have sought to adapt, extend and update an existing Institute for Government timeline of attempts to join up public services at a local level in England (Wilson et al, 2015). This new dataset (Annex II) organises and systematises information about different central approaches to joining up public services, which will help make sense of the often confusing, overlapping, and competing strategies that underpin the approach to securing joined-up services (see Hood, 2005).

To identify a comprehensive set of centrally-initiated joining up initiatives, we develop formal inclusion criteria (see first subsection in the fourth section). These criteria were used alongside a detailed search of central government websites and archives (the National Archives’ UK Government Web Archive, GOV.UK and Public Information Online) to identify relevant initiatives introduced since the publication of the original dataset. We also used these criteria to corroborate the suitability of programmes included in the preceding report (Wilson et al, 2015). The list of initiatives was validated through correspondence and discussions with senior civil servants from a range of central government departments who are involved in contemporary joining-up initiatives, and social policy experts who have conducted similar reviews in the past. Nevertheless, it may be incomplete. There is no centralised repository documenting government policy initiatives, and no single way in which these initiatives are announced – some pass through Parliament as bills, others are presented within white papers, spending reviews or budgets, while others still are announced in individual press releases. However, the dataset which underpins this article represents the most comprehensive and up-to-date dataset of central government initiatives to join up local public services in England.^2^

We conducted qualitative content analysis to analyse the three dimensions of the mechanism of central to local join-up presented earlier (in the third section), and describe the similarities and variations across initiatives. We adopt a directed approach (Hsieh and Shannon, 2005), also referred to as deductive category application (Mayring, 2000), developing and applying a formal coding framework (second subsection of the fourth section; for full framework, see Annex I) which operationalises established theoretical dimensions. We apply this framework to all initiatives identified between 1997 and February 2022 (n=55). This allows us to describe the characteristics of different initiatives, and map the frequency of different approaches, as well as variation over time.

### Inclusion criteria and search strategy

The focus of this work is to understand how central government might better support the coordination of local public services. As such, to be included in this dataset, initiatives (or substantive sub-themes within broader initiatives) must express three characteristics. Included initiatives must:

1) be instigated by central government, either from within a central spending department or from a dedicated, centralised policy function in Whitehall;
2) articulate an intention to reshape (or otherwise alter) the provision of complex, person-facing cross-cutting public services. These are services focused on people and require coordination across administrative boundaries either vertically or horizontally, sometimes within the public sector, sometimes spanning the private and social sectors.
  The language around coordination and joining up has changed over time, and so in some instances we have included projects that use more general terms such as ‘transformation’ or ‘delivering differently’ (particularly where this is mentioned in relation to cross-government or cross-sector delivery arrangements);
3) aspire, either explicitly or implicitly, to change the nature of services at a subnational level in England. Initiatives may target different sub-national ‘units’, from regional to local authority to neighbourhood level.

Individual initiatives, which are each included as a row in the dataset, are distinguished based on distinct, ‘announceable’ projects at a central government level. This is based on researcher interpretation of a range of factors, including whether an initiative was named, whether it had different eligibility criteria or substantive features to a related initiative, and whether it targeted more than one local area. For example, while the formation of a Local Strategic Partnership (LSP) was a requirement for receipt of money from the Neighbourhood Renewal Fund (NRF), the two initiatives were distinctly named, and only those LSPs covering the 88 most deprived neighbourhoods were eligible for NRF funding. As a result, we classify these as two distinct initiatives. Conversely, while individual City and Devolution deals have been struck on an *ad hoc* basis and so have different arrangements, they are all part of broader, centrally named initiatives covering multiple localities, and so ‘City Deals’ are listed as a single initiative in our dataset. As many initiatives also cover a broader set of policies, we focus only on the content related to the joining up of complex public services.

The volume of accessible documentary information available on initiatives varies significantly, from one or two policy announcements or archived webpages to large scale, independent programme evaluations. To enable relatively consistent data extraction for each programme, we attempted to identify two documents for each initiative, although on some occasions only one document was available. Where possible, at least one document was an official government document, such as a white paper, policy announcement, or official evaluation.^3^ Other documents include academic literature and independent commentary.

### Coding framework

To analyse these documents, we developed a structured coding framework using a mix of two coding approaches. First, we use attributive coding to systematically extract and organise high-level information from the documents, such as the start date and end date of a programme (if available) and the central department(s) responsible for particular programmes. Attributive coding helps ‘fix an early structure for the data set establishing a good overview and easy access to data’ (Linneberg and Korsgaard, 2019). Attributive coding also helps to identify potential overlaps in programme launch dates and departmental ownership. Clear labelling of key dates allows us to identify variation in approach that may correspond to temporally bounded factors, such as the political administration of the day.

Second, we develop and apply a mutually exclusive coding framework (Schreier, 2014). In accordance with deductive category application (Mayring, 2000), this is underpinned by the theoretical frameworks described earlier, in the third section, to describe three features of each initiative: the central government lever used to initiate joining up; the allocation process used to decide which areas or populations will benefit; and the role that local actors are intended to play in the initiative. The coding framework for the three substantive dimensions is included in Annex I. Coding of all documents was undertaken by a single lead researcher. In order to ensure reliability, a sample of initiatives were audited by two additional coders. Any unclear cases and discrepancies were then taken to a reconciliation meeting, where they were discussed and resolved.

## Results

### How has central government attempted to use its levers to join up public services at a local level?

**Figure 1:**
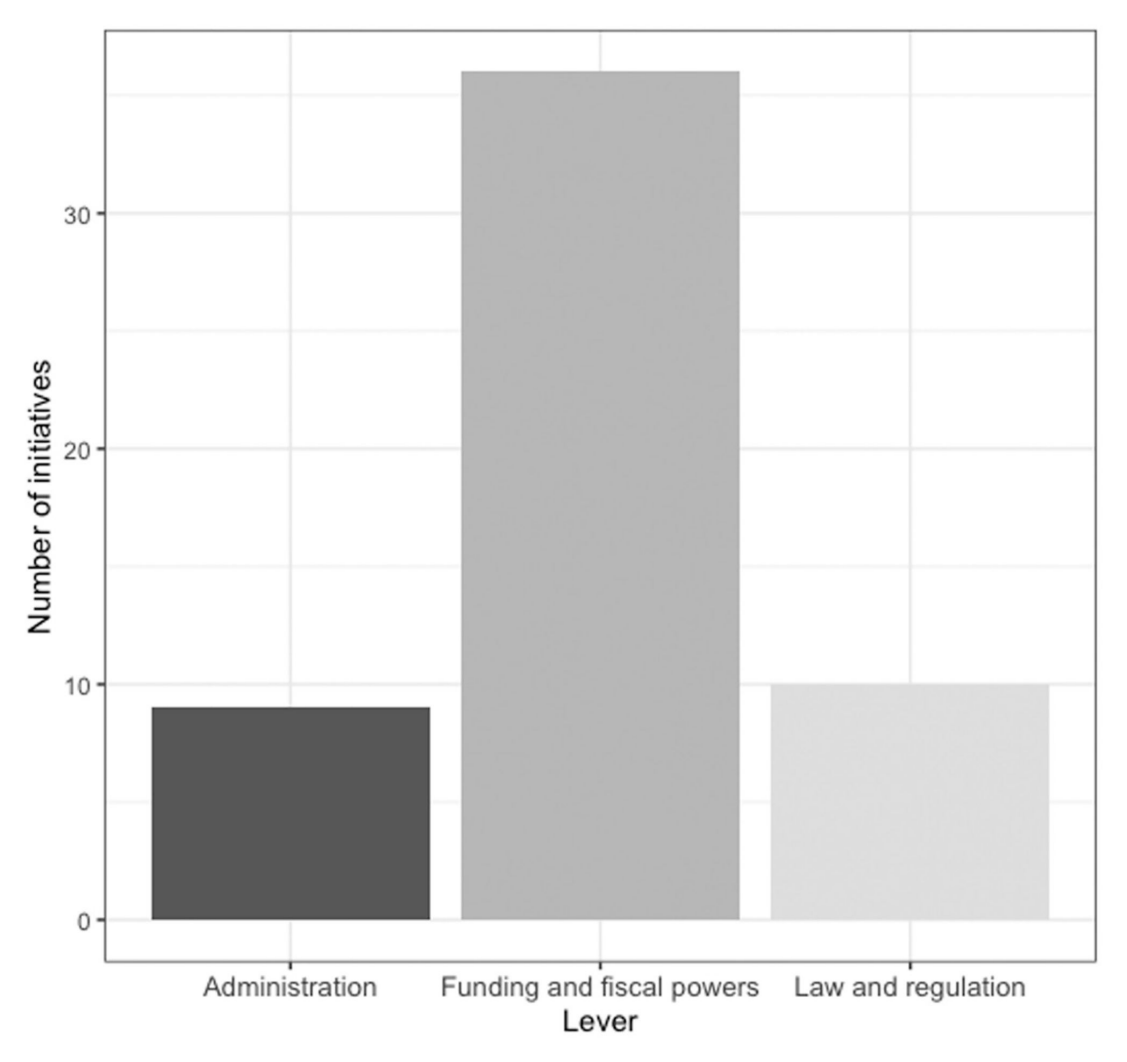
Lever for local joining up initiatives, 1997–2022

Over the last 25 years, Westminster government has used law and regulation, funding and fiscal powers, and administrative reforms to join up local public services through at least 55 distinct initiatives. However, as Figure 1 shows, despite having a number of levers at its disposal with which to affect these reforms, it has overwhelmingly relied on the use of funding and fiscal powers (36). In comparison, administration (9) and law and regulation (10) have been largely neglected as tools to improve local coordination.

**Figure 2:**
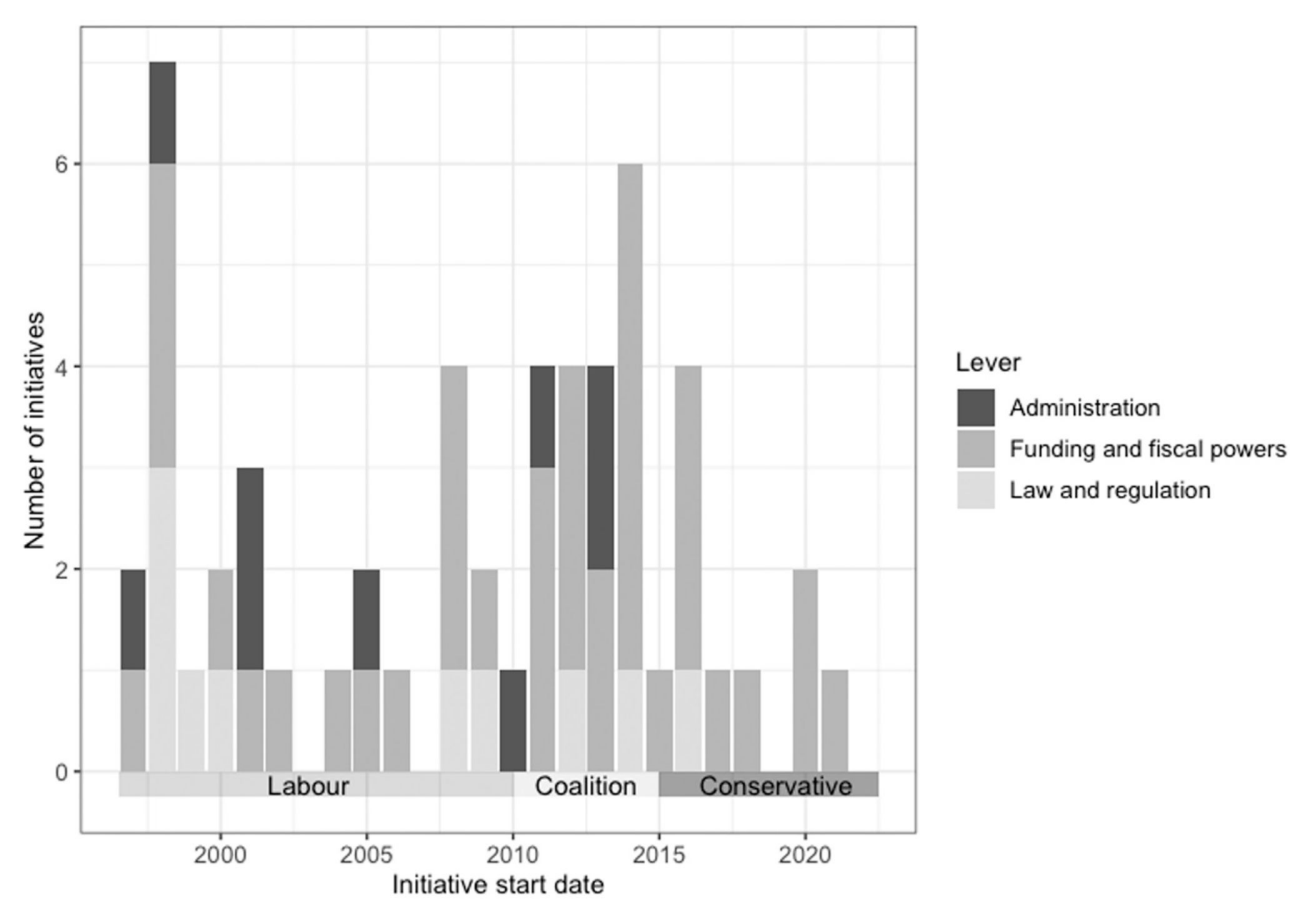
Lever over time

Figure 2 shows the lever used in each initiative over time, based on the start date of each initiative. The use of funding as the main lever to join up services appears to have remained relatively consistent over the period under study. Nevertheless, funding represents the only lever used among the small group of initiatives which started in the last five years.

### How have the recipients of joining up been selected?

**Figure 3:**
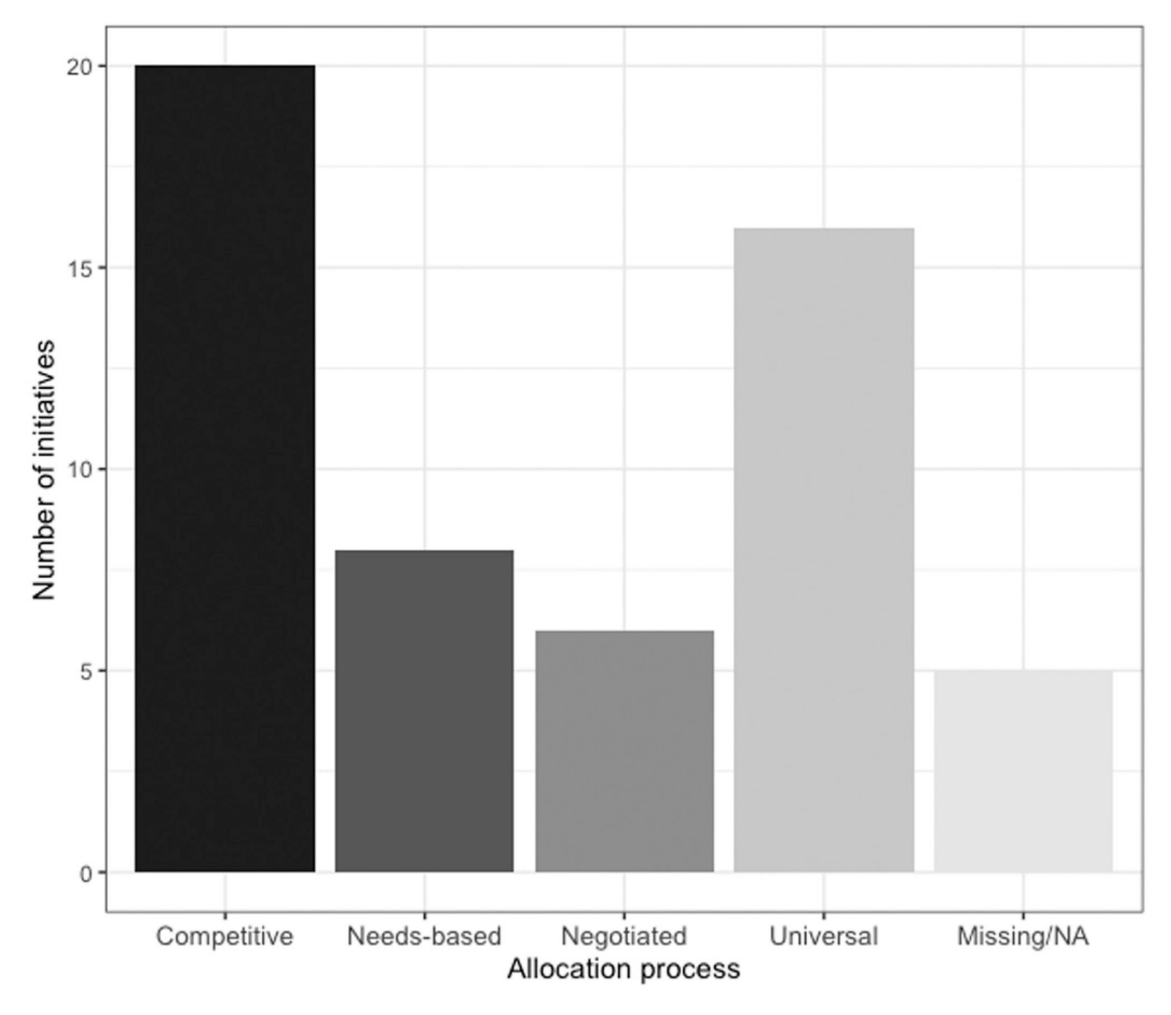
Allocation process of local joining up initiatives, 1997–2022

We examined the process used by central government to decide which places would benefit from the initiative (Figure 3). The most common allocation process used was a competitive one (20), in which areas bid against each other, and those with the highest scoring bids were allocated to the initiative. The use of competition is particularly striking when the focus is narrowed to targeted allocation processes, ignoring universal initiatives (16) which represent the second most common allocation process. Other allocation processes – needs-based (8) and negotiated (6) – were used relatively infrequently. Finally, five initiatives either lacked information on the allocation process used, or could not be said to have been ‘allocated’ to places – for example, the Social Exclusion Unit was a central government coordination advisory unit that did not target specific local areas.

**Figure 4:**
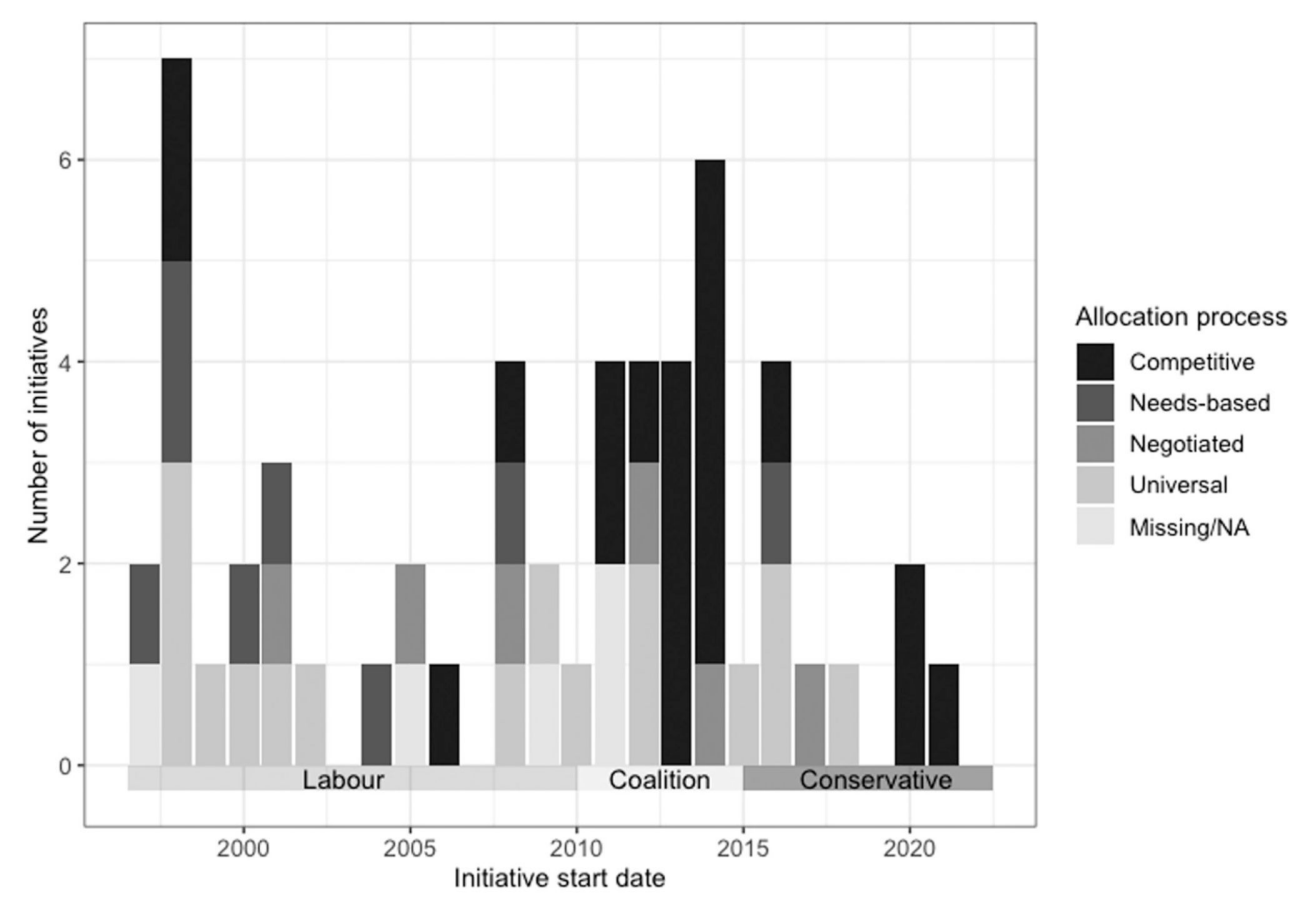
Allocation process over time

Figure 4 shows the allocation process used by each initiative, plotted according to the start date of the initiative. Prior to 2010, the most common targeted allocation process was needs-based, in which areas receive support according to their perceived need. However, after 2010, the majority of initiatives are allocated according to a competitive process, in which areas must submit bids and compete with one another to receive support. This shift coincides with a shift in government, from a Labour administration to a Conservative–Liberal Democrat Coalition (in which the Conservative party were the major partner). This era of competition also aligned with a period of austerity in public spending, introduced by the Coalition government in response to the 2008 financial crisis and maintained by subsequent Conservative administrations.

### What role(s) has central government envisioned for local actors in joined-up local public services?

Often, the rhetoric surrounding local joining-up initiatives has been closely aligned with that of devolution and autonomy for local institutions. One might therefore expect that joining-up initiatives would largely follow a community leadership model, in which local institutions are responsible for setting the objectives of initiatives within their area, and hold the delivery of the initiative to account for achieving those locally designed objectives.

**Figure 5:**
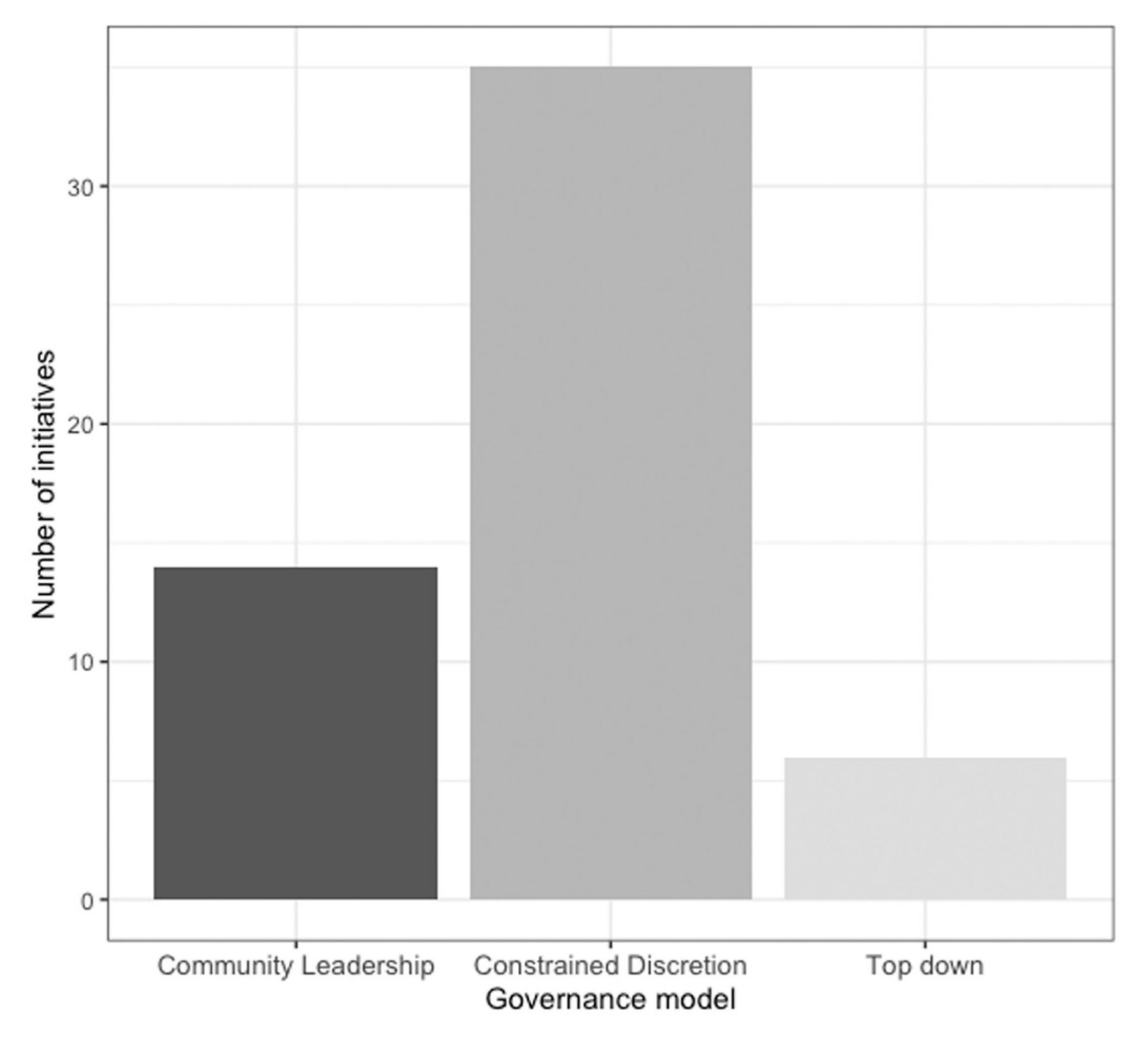
Governance model of local joining-up initiatives, 1997–2022

However, as Figure 5 demonstrates, the dominant model has not been one of wholesale localisation of accountability. Instead, central government has tended to opt for a constrained discretion model, in which autonomy over delivery is combined with central control over the objectives that are being pursued, and accountability to the centre for whether they are achieved. Perhaps even more striking given the aforementioned rhetorical alignment between devolution and joining up, some initiatives displayed a top-down governance model, in which even the approach to delivering the initiative was tightly prescribed by central government.

**Figure 6:**
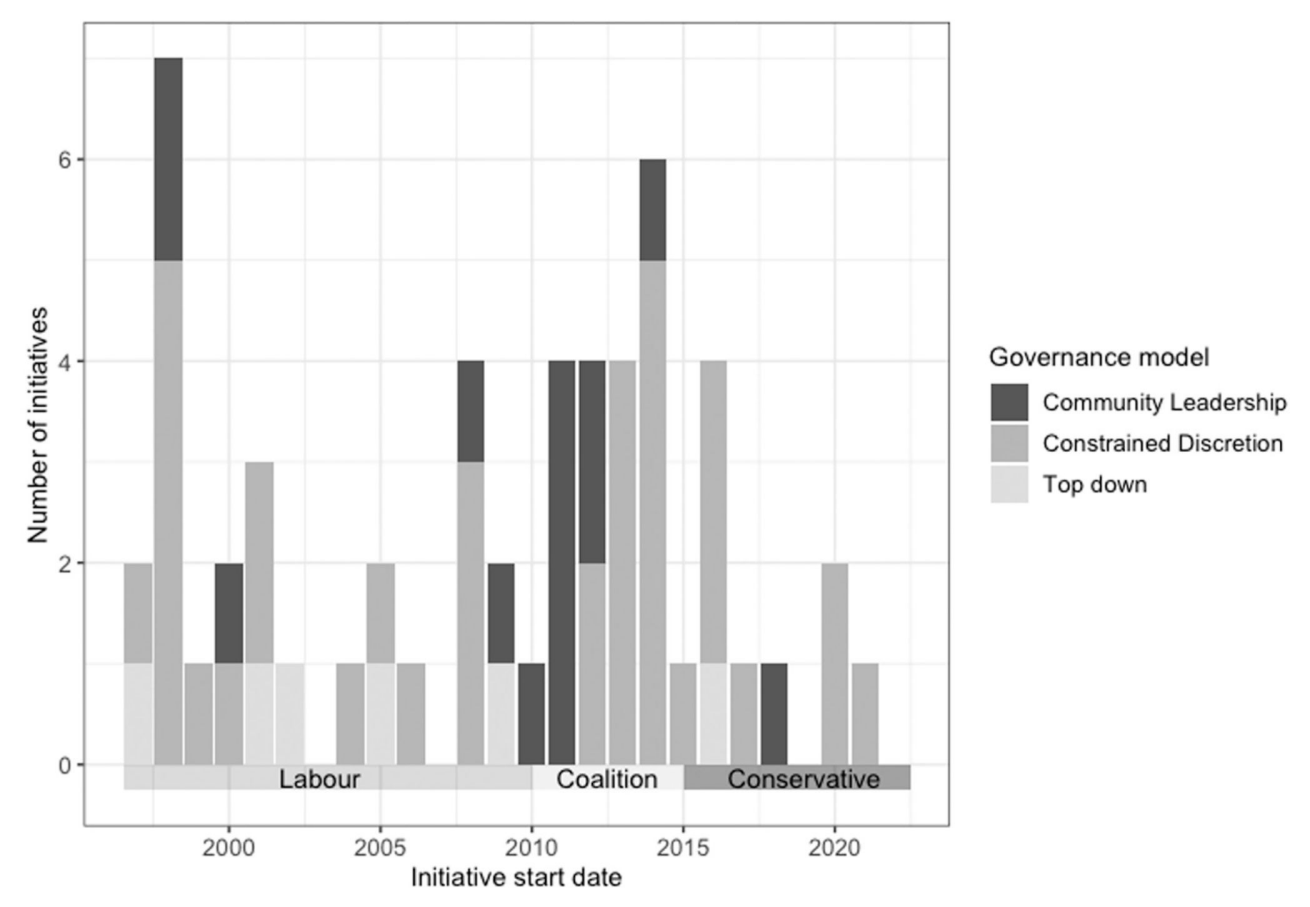
Governance model over time

Nevertheless, Figure 6 suggests this has not been wholly consistent over time. Between 2010 and 2012, we see a cluster of initiatives which appear to pursue a community leadership model of governance, in which local actors take on responsibility for objective setting and accountability in addition to delivery approach. This timing seems to align with the Open Public Services agenda of the coalition Conservative–Liberal Democrat government, which held as its second principle that ‘power should be decentralised to the lowest appropriate level’ (HM Government, 2011). Whether this agenda did in fact mark a shift in central–local relations, and why it failed to achieve longer term impact beyond 2012 (after which the more general trend of constrained discretion continued) may provide an interesting avenue for future research.

## Discussion and conclusions

Over the last 25 years, central government has attempted to join up local public services in England on at least 55 occasions, illustrating the ‘initiativitis’ inflicted upon local governments by the large volume and variety of coordination programmes (Stoker, 2005: 159; 6 et al, 2002). By analysing and mapping some of the characteristics of these initiatives, we have uncovered insights into the ways central government has sought to achieve local coordination. We observe a clear preference for the use of funding and fiscal powers as a lever, a competitive allocation process, and a constrained discretion model of governance, with some distinct patterns over time. These choices made in the design of initiatives are likely to be shaped by the perceived and real accountability structures within government, and so offer an opportunity to consider how accountability affects, and is affected by, particular programmatic efforts at a local level.

In the first subsection of the fifth section, we highlighted the overwhelming reliance on the use of funding and fiscal powers to achieve local joining up. Often, this is part of a package of new central funding for local actors to address cross-cutting social issues in a more coordinated way. For example, the Troubled Families programme (now known as Supporting Families) has provided over £100 million annually over the last ten years for local authorities to provide support to families facing multiple and complex disadvantages related to crime and antisocial behaviour, education, life chances, living standards, domestic abuse and mental and physical health (Loft, 2020). In other initiatives, there was no ‘new’ money as such. Instead, the government employed various kinds of ‘financial acrobatics’, with an emphasis on restructuring existing funding at the local level to achieve greater coordination. Generally, this took the form of mapping public spending and attempting to create ‘pooled budgets’, in which traditionally separate national and local funding streams would be pooled into a single, flexible local budget, as in Total Place and the various Community Budget pilots which followed.

In comparison, administration and law and regulation have been used relatively sparingly. Nevertheless, they have made notable contributions to the landscape of joining-up attempts. Legislation has underpinned some of the most substantial reforms, including Regional Development Agencies and devolution deals, as well as some of the longest lasting, such as Youth Offending Teams and Crime and Disorder Reduction Partnerships. Similarly, while the need for resources to achieve coordination should not be underestimated (Kania and Kramer, 2011), administrative reforms using reorganisation and persuasion can be valuable in establishing structures that cut across individual funding initiatives.

Meanwhile, in the the third subsection of the fifth section, we observe a clear tendency towards a constrained discretion model of governance, in which despite some flexibility around delivery, the centre retains control over the programme’s objectives and remains the ultimate locus of accountability. Discovering that England is a centralised administrative landscape is not in itself particularly novel (for example, Pike et al, 2020). However, our findings provide a further empirical basis for a much-repeated claim. Our analysis highlights the stark reality that, even in some of the most explicit efforts to decentralise control and join up local public services, central government has largely been unable or unwilling to pass the reins of accountability down to the local level. Despite broad and vocal support for localism, in both politics (HM Government, 2011; DLUHC, 2022; Commission on the UK’s Future, 2022) and policy analysis (for example, Powell and Boyne, 2001; although see Fitzpatrick et al, 2020; Collier, 2020), practice – at least in terms of initiatives which seek to better coordinate local public services – appears much more muted.

This accords with an approach to policy design and management which is dominated by the Westminster model of accountability, in which accountability must ultimately run through ministers to parliament (Barberis, 1998). At least in part, the emphasis on a constrained discretion model may be due to the inherent limitations associated with the levers used by central government to enact these initiatives. As discussed earlier, the majority of initiatives involve the spending of centrally-held public money, which is subject to parliamentary oversight. Thus, without appropriate structures in place, it may be difficult for central government to entirely devolve accountability. If a government minister is liable to be called in front of a select committee or to the dispatch box to answer for performance or value for money, it is perhaps inevitable that they will be unwilling to relinquish (the perception of) control without significant reassurances. When this accountability balancing-act is pursued via a constrained discretion model, central accountability is accompanied by local adaptability. While this may superficially appear to strike a compromise, the decoupling of control and responsibility risks muddying the waters of accountability (Mulgan, 2014). When initiatives are successful, everyone is happy to claim their share, but when things go wrong, this arrangement risks a descent into buck-passing.

This points towards the need to explore alternative models of accountability, which tackle head on the multi-level nature of these initiatives. Despite the dominance of constrained discretion, at two temporal extremes of our study, the New Deal for Communities (NDC) and Integrated Care Systems (ICSs) provide rare examples of central funding initiatives that nonetheless appear to pursue a community leadership model. While further analysis would be required to offer concrete conclusions as to why this may be the case, both created specific governance boards – in the case of the NDC ‘Partnership Boards’, and ICSs ‘Integrated Care Boards’ – as a means of local oversight. These pioneering governance approaches offer examples of ‘Type II’ multi-level governance, in which structures are established to govern particular task-focused jurisdictions (Hooghe and Marks, 2003). In the context of a hollowed-out local government that may be perceived to lack the capacity to provide sufficient local scrutiny of initiatives, it may be that these new local governance and accountability instruments are required to facilitate greater local autonomy.

Type II governance has two key potential drawbacks, however. The establishment of governance arrangements which have an unclear relationship to the hierarchy risks creating a de jure accountability deficit (Mulgan, 2014). While boards may (and often do) include representatives of both central and local government, and Westminster politicians are likely to be held de facto accountable for its success or failure, they generally do not hold direct authority over the programme (Mulgan, 2014). Second, in the context of efforts to better coordinate public services, a Type II approach risks simply replacing a patchwork of ‘traditional’ policy silos with a new patchwork of governance silos, albeit potentially more internally coherent ones.

How, then, might central government pursue more effective local accountability? An alternative approach is offered by Type I multi-level governance, which is implemented through durable, general purpose functions (Hooghe and Marks, 2003). If something akin to the devolved administrations in Scotland, Wales and Northern Ireland were replicated in English regions, with clear democratic accountability and broad powers over public services and public finances, then the need for the centre to drive local coordination would be diminished. This would solve the two challenges identified with the pursuit of Type II governance, establishing more localised and coherent de jure accountability structures, but would mark a much more significant departure from the Westminster model. Our analysis suggests a limited appetite for such a shift thus far, with prototype examples such as devolution deals rare.

As it stands, central government retains ultimate democratic responsibility for the success or failure of public service provision. While this remains the case, even if the centre believes that local autonomy is desirable, it will wish to maintain some form of oversight, maintaining the tensions discussed earlier. However, with both major political parties seemingly in favour of greater local autonomy (DLUHC, 2022; Commission on the UK’s Future, 2022), there is much scope for further empirical work to understand how this can be reconciled with a tradition of centralised accountability.

## Next steps

### Limitations of the analysis

There are a number of limitations to the analysis presented earlier. We have extracted limited details on the initiatives, and in particular, lack detail on their scale – in terms of time, magnitude of funding, geographic coverage (beyond a simple distinction between universal and targeted initiatives), and their substantive policy scope. The analysis is also limited to the *intention* of the various initiatives. We do not explore whether the initiatives in practice lived up to their intention on paper. For example, an initiative that aspires to genuinely devolve objective setting and accountability may not do so in practice. The *ex-ante* focus also means we are not able to comment on the impact of these initiatives. Because of this, we are not able to assess whether particular initiatives are more effective, or whether configurations of the dimensions explored here are more or less likely to lead to greater coordination.

In addition, our study of joining up policy cannot serve as a standalone lens into central–local relations. Joining-up initiatives that focus on complex, cross-cutting person-facing services represent only one aspect of the interaction between central government and local institutions, in terms of both scope and monetary value. Local institutions, and in particular local government, receive a range of direct funding from central government, of which the initiatives described here are likely to represent only a small part. Our focus on joining up cannot cover the whole range of public service reform. Coordination reforms tend to target particular elements of public services, namely those that deal with cross-cutting issues. However, much of the interaction between central and local government over the period of our study has concerned more mainstream elements of public services, including health and education. Finally, our analysis should be read against the context of over a decade of austerity, which has seen dramatic cuts to local public service funding (Ogden et al, 2021). While many of the individual initiatives explored in this work ostensibly intend to give greater funding and autonomy to local institutions, this must be viewed against a backdrop of macro-scale cuts to local funding from central government in the aftermath of the 2008 financial crisis.

Joining up is still a valuable lens, but it is nevertheless important to also see the wider context in which it sits. Some of this may be addressed by future extension of this work, by extracting further data from the policy documents already collected, supplemented with additional material, or by adapting the analysis to other areas, such as core local government services and funding, or devolution of powers more broadly.

### A changing policy context – levelling up

At the heart of the current government’s flagship levelling up agenda is an ambition to address the impact of spatial inequalities on both social and economic outcomes. Some inequalities may be ameliorated by hard infrastructure, but others will require tackling social problems. The challenges outlined at the beginning of this article are often felt most acutely in ‘left behind’ places, where generally lower public service capacity interacts with higher rates of multiple and complex disadvantage (Atkins and Hoddinott, 2022). Future trends remain unclear, although there were some early signals offered in the government’s Levelling Up white paper, which laid out a series of programmes and policies to take forward the agenda (DLUHC, 2022). Of particular interest is its stated ambition for ‘every part of England that wants one’ to have a comprehensive devolution deal and simplified, long-term funding settlement (DLUHC, 2022: 121). The implication of this, as laid out in the accompanying devolution framework, is a push towards more directly elected mayors to fulfil the governance role that appears important for community leadership. It will be important to consider how public accountability may evolve in the context of further and wider devolution, and how the findings presented earlier may inform these developments.

### Future research directions

This analysis offers emerging insights into efforts to join up local complex public services, and broader relationships between central government and local institutions in England, laying the groundwork for several avenues of future research.

There is an opportunity to further extend the dataset we have built, in order to better understand the extent to which local joining-up initiatives have been successful. As new joining-up initiatives are launched in the context of the levelling up agenda, there will be opportunities to extend the dataset. Equally, while the election of the New Labour administration in 1997 – and its accompanying focus on tackling complex social problems through joining-up initiatives – marks an obvious starting point for the dataset, there may be reasons to explore earlier initiatives. And while our analysis is limited to England, there may be valuable insights to be found in expanding the geographical scope to the rest of the UK, or indeed further afield. Building on this expanded dataset, analysis should focus on exploring initiatives in more depth. We focus on a limited set of elements describing programme intent. There is, therefore, significant scope to capture more details on the characteristics of initiatives as implemented, and ultimately, on the outcomes achieved as identified by evaluation material. This would allow us to not only assess the success of individual initiatives, but also to test an implicit assumption that underpins the scope of this analysis, as well as much of the policy and academic approach to improving policy coordination in recent decades – that it is ‘easier to secure joined-up government at the local level than in Whitehall’ (Bogdanor, 2005: 12).

The enduring requirement for central accountability in England is also a spur to further research. Our findings illustrate the apparent unwillingness or inability of central government to truly devolve power and accountability to local institutions, an issue long-discussed and critiqued, most recently in an inquiry of the Public Administration and Constitutional Affairs Committee (PACAC, 2022). However, our dataset also reveals several initiatives that attempt to navigate centralised accountability structures in different ways, and valuable research could be conducted on the mechanisms through which accountability structures can be re-formed and enabled. One line of inquiry could investigate the proposed large-scale accountability shifts created by devolution deals and their impact on joined-up service delivery, complementing and broadening emerging insights on the relationship between increased decision-making and budgetary powers and improved cross-sector collaboration (Britteon et al, 2022). Another could focus on more programme-specific mechanisms designed to create new structures for accountability, for example by mediating the need for central control via outcomes frameworks. Such frameworks are assumed to be helpful mechanisms to align and coordinate service delivery along a common set of strategic goals, while remaining agnostic around *how* such coordination is implemented at the local level (Wimbush, 2011; Savell et al, 2021).

The analysis presented in this article, and that proposed earlier, might also be supplemented with explorations of other instances of central–local relations, such as through wider funding relationships or governance structures – we have only elaborated on a few examples of research avenues which our dataset enables. However, we are confident that it will serve as a springboard for further research which might take the lessons from a bad case of ‘initiativitis’ and apply them to a more coordinated agenda to address the fragmentation of accountability and service provision in England.

## Figures and Tables

**Table 1:** Tools of government to central government levers, adapted from Hood and Margetts, 2007

Hood and Margetts resource	Hood and Margetts description	Central government lever
Authority	Authority denotes the possession of legal or official power (Lasswell and Kaplan, 1950:76, n. 2). That is the power officially to demand, forbid, guarantee, adjudicate.	Law and regulation
Treasure	Treasure denotes the possession of a stock of moneys or ‘fungible chattels’. That means not only (or necessarily) money in the common, everyday sense of banknotes or coins, but anything which has the money-like property of ‘fungibility’ (Rose and Peters, 1978: 25) – that is, the capacity to be freely exchanged.	Funding and fiscal powers
Nodality	Nodality denotes the property of being in the middle of an information or social network (not necessarily ‘dead centre’) : the ‘coin’ – how government spends this resource – is messages sent and received.	Administration
Organisation	Organisation denotes the possession of a stock of people with whatever skills they may have (soldiers, workers, bureaucrats), land, buildings, materials, computers and equipment, somehow arranged.	

**Table 2:** Dimensions of governance models, adapted from Stoker (2005)

	Community leadership	Constrained discretion	Top down
**Objective setting (political) accountability**	Local	Central	Central
**Approach to achieving objectives**	Local	Local	Central

## Notes

1 The picture in the rest of the United Kingdom for most of the period of our study is further complicated by another layer of government, the devolved administrations in Scotland, Wales and Northern Ireland. As a result, we focus on initiatives within England (although some initiatives may have been implemented more widely within the UK).

2 This dataset also underpins a live, interactive policy tool which allows users to navigate the evidence and insights from these initiatives. This can be found at https://golab.bsg.ox.ac.uk/knowledge-bank/indigo/joining-up-services/

3 Information from evaluation documents was extracted from the descriptive background portion of the article, rather than the substantive evaluation portion.
